# Selection of trustworthy crowd workers for telemedical diagnosis of pediatric autism spectrum disorder

**Published:** 2021

**Authors:** Peter Washington, Emilie Leblanc, Kaitlyn Dunlap, Yordan Penev, Maya Varma, Jae-Yoon Jung, Brianna Chrisman, Min Woo Sun, Nathaniel Stockham, Kelley Marie Paskov, Haik Kalantarian, Catalin Voss, Nick Haber, Dennis P. Wall

**Affiliations:** 1 Department of Bioengineering, Stanford University, Palo Alto, CA, 94305, USA; 2 Department of Pediatrics (Systems Medicine), Stanford University, Palo Alto, CA, 94305, USA; 3 Department of Biomedical Data Science, Stanford University, Palo Alto, CA, 94305, USA; 4 Department of Computer Science, Stanford University, Palo Alto, CA, 94305, USA; 5 Department of Neuroscience, Stanford University, Palo Alto, CA, 94305, USA; 6 School of Education, Stanford University, Palo Alto, CA, 94305, USA

**Keywords:** Crowdsourcing, Machine Learning, Diagnostics, Trust, Privacy, Autism

## Abstract

Crowd-powered telemedicine has the potential to revolutionize healthcare, especially during times that require remote access to care. However, sharing private health data with strangers from around the world is not compatible with data privacy standards, requiring a stringent filtration process to recruit reliable and trustworthy workers who can go through the proper training and security steps. The key challenge, then, is to identify capable, trustworthy, and reliable workers through high-fidelity evaluation tasks without exposing any sensitive patient data during the evaluation process. We contribute a set of experimentally validated metrics for assessing the trustworthiness and reliability of crowd workers tasked with providing behavioral feature tags to unstructured videos of children with autism and matched neurotypical controls. The workers are blinded to diagnosis and blinded to the goal of using the features to diagnose autism. These behavioral labels are fed as input to a previously validated binary logistic regression classifier for detecting autism cases using categorical feature vectors. While the metrics do not incorporate any ground truth labels of child diagnosis, linear regression using the 3 correlative metrics as input can predict the mean probability of the correct class of each worker with a mean average error of 7.51% for performance on the same set of videos and 10.93% for performance on a distinct balanced video set with different children. These results indicate that crowd workers can be recruited for performance based largely on behavioral metrics on a crowdsourced task, enabling an affordable way to filter crowd workforces into a trustworthy and reliable diagnostic workforce.

## 1. Introduction

Autism spectrum disorder (ASD, or autism) is a pediatric developmental condition affecting 1 in 40 children in the United States [1], with prevalence continuing to rise [2]. While access to care relies on a formal diagnosis from a clinician, an uneven distribution of diagnostic resources across the United States contributes to increasingly long waitlists. Some evidence suggests that 80% of counties lack sufficient diagnostic resources [3], with underserved communities disproportionately affected by this shortage [4]. Telemedicine has the potential to minimize this gap by capitalizing on the increasing pervasiveness and affordability of digital devices. Such diagnostic solutions are especially pertinent during times of pandemic, most notably the coronavirus, which further hinders access to diagnosis and care.

Mobile digital autism interventions administered on smartphones [5-12] and on ubiquitous devices [13-27] passively collect structured home videos of children with neuropsychiatric conditions for use in subsequent diagnostic data analysis [27-28]. In order for the video data collected from digital therapies to become widely used, trustworthy data sharing methodologies must be incorporated into the diagnostic pipeline [29]. One possible approach, which we realize in the present study, is to carefully recruit a trustworthy set of workers to transform the video streams into a secure, quantitative, and structured format. While modern computer vision algorithms could handle this task in several domains, extracting complex behavioral features from video is currently beyond the scope of state-of-the-art machine learning methods and therefore requires human labor. However, the collected videos naturally contain highly sensitive data, requiring careful selection of trustworthy and reliable labelers who are allowed access to protected health information (PHI) after completion of Health Insurance Portability and Accountability Act (HIPAA) training, Collaborative Institutional Training Initiative (CITI) human subjects training, and whole disk encryption.

In the present study, we examine strategies for quantitatively determining the credibility and reliability of crowd workers whose labels can be trusted by researchers. It is important that the metrics for evaluating workers are speedy and simple, as formally credentialing recruited crowd workers through institutional channels is laborious and slow. We crowdsource the task of providing categorical feature labels to videos of children with autism and matched controls. For each crowdsourced worker, we evaluate correlations of their mean classifier probability of the correct class (PCC) using their answers as input with (1) the mean L1 distance between their responses to the same video spaced one month apart, (2) the mean L1 distance between their answer vector to each video and all other videos they rated, (3) the mean time spent rating videos, and (4) the mean time and L1 distance of answers when the worker is explicitly warned about not spending enough time rating a video and provided with a chance to revise their response. We then feed the metrics which are correlated with PCC into a linear regression model predicting the PCC.

## 2. Methods

### 2.1. Clinically representative videos

We used a set of 24 publicly available videos from YouTube of children with autism and matched neurotypical controls (6 females with autism, 6 neurotypical females, 6 males with autism, and 6 neurotypical males). Criteria for video selection and inclusion were that (1) the child’s hand and face must be visible, (2) opportunities for social engagement must be present, and (3) an opportunity for using an object such as a toy or utensil must be present. Child diagnosis was determined through the video title and description. The videos were short, with a mean duration of 47.75 seconds (SD = 30.71 seconds). The mean age of children in the video was 3.65 years (SD = 1.82 years).

### 2.2. Crowdsourcing task for Microworkers

Prior work has validated the capability of subsets of the crowd recruited from the Amazon Mechanical Turk crowdsourcing platform [30] to provide feature tags of children with autism comparable to clinical coordinators working with children with autism on a daily basis [31-32]. We instead recruited workers from Microworkers.com, as Microworkers consists of a diverse representation of worker nationalities [33] compared to Mechanical Turk, which contains workers mostly from the United States and India [34]. Furthermore, Microworkers provides built in functionality for allowing workers to revise their answers if a requester is unsatisfied but believes the worker can redeem their response. This functionality was crucial for our trustworthiness metric.

**Fig. 1.**
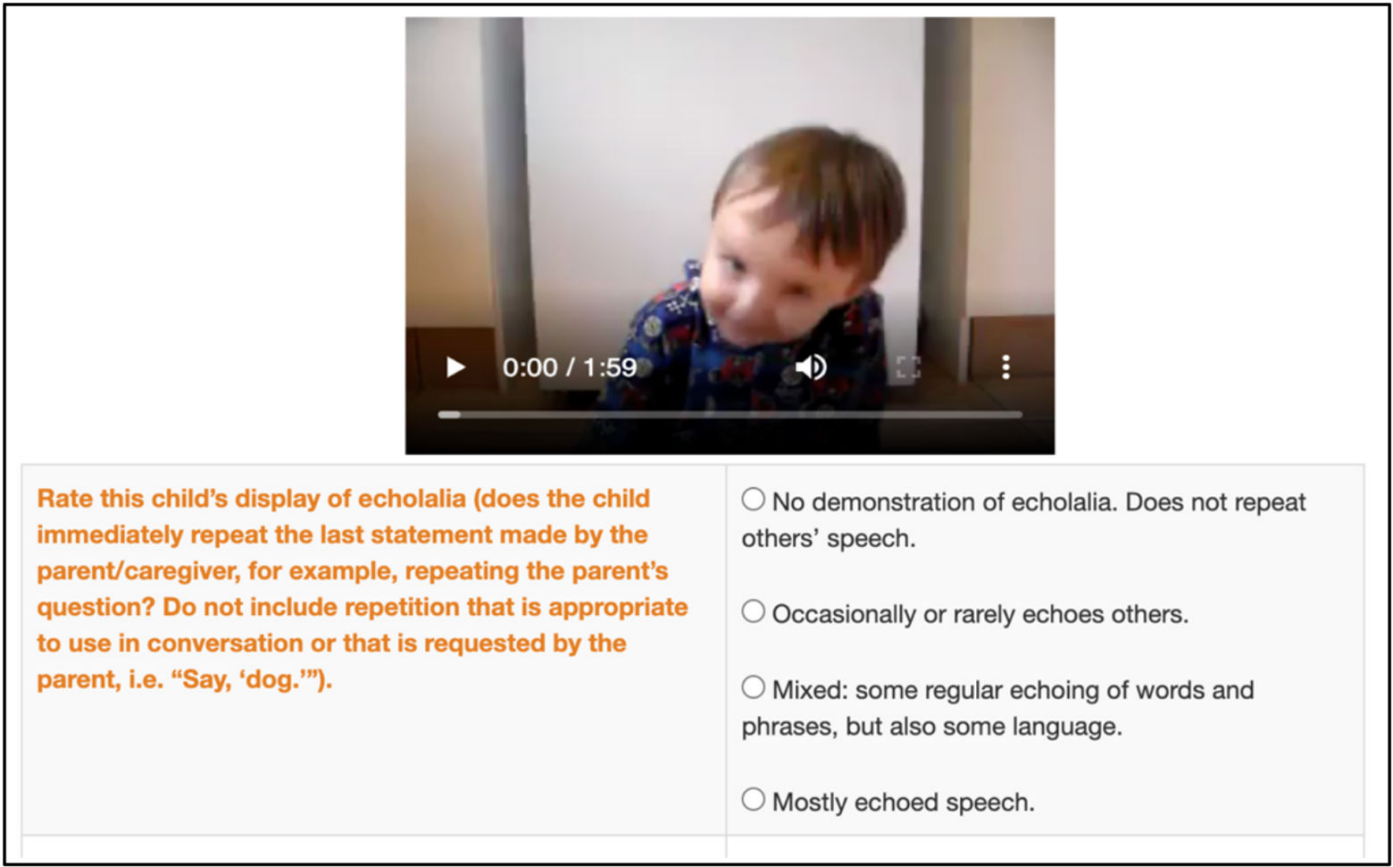
Crowd worker feature tagging user interface deployed on Microworkers.com. Each worker answered a series of multiple-choice questions corresponding to each input feature of a gold standard classifier.

The task consisted of a series of 13 multiple choice questions identified, in prior work which employed feature selection algorithms on electronic health records [35-44], as salient categorical ordinal features for autism prediction. Workers were asked to watch a short video and answer the multiple-choice questions using the interface depicted in Fig. 1. Microworkers automatically records the time spent on each task.

Through a pilot study of internal lab raters providing 9,374 video ratings for which we logged labeling times, we observed that the mean time per video was 557.7 seconds (9 minutes 18 seconds), with a standard deviation of 929.7 seconds (15 minutes 30 seconds). The pilot task consisted of answering 31 multiple choice questions, while the Microworkers task only contained 13 questions; the proportional mean time is 233.9 seconds (3 minutes 54 seconds). We therefore required workers to spend at least 2 minutes per video, a time threshold significantly below the 233.9 second mean proportional time. If any crowd worker spent less than 2 minutes rating a video, we leveraged the built-in functionality on Microworkers to prompt these users to revise their answers and sent them a warning message disclosing that we know the “*Impossibly short time spent on task*.” We measured the additional time spent by the worker, if any, as well as the changes in the answer vector (L1 distance) after receiving this message.

We posted all tasks for all 24 videos exactly 30 days after the original task, allowing workers who completed the first task to complete the task again while minimizing the chance that they could use the memory of their prior responses to bias the test. Previous studies which evaluate test-retest reliability consider 2 weeks to be sufficient time to prevent memorization of prior administrations of the questionnaire [45-48], and we increased this time frame to 30 days to minimize the likelihood that any memory of the workers’ previous answers remained. The same video of the child was provided for both administrations of the task. Workers were not provided with their original answers for reference. The difference between the worker’s original answers and their revised answers on the same video served as quantitative information about the *reliability* of the worker.

### 2.3. Classifier to evaluate performance

**Fig. 2.**
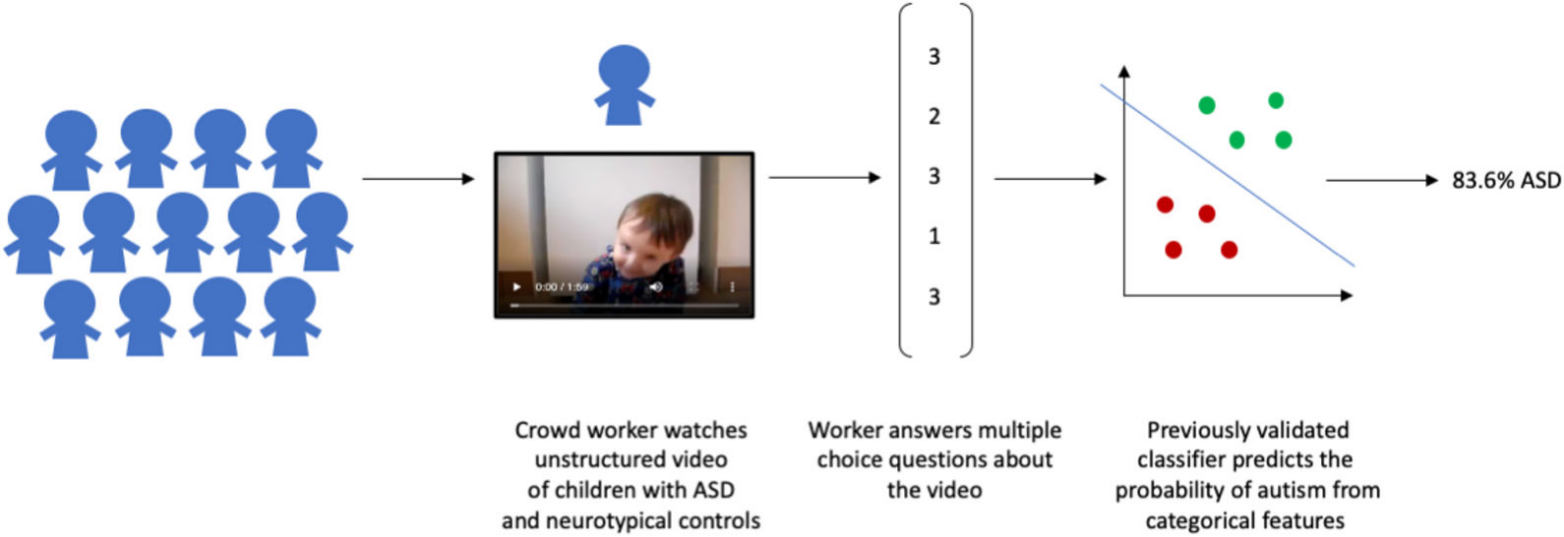
Process for collecting the data needed to evaluate trust and reliability metrics for crowd workers. Each crowd worker watches unstructured videos of children with autism and neurotypical controls, answering multiple choice questions about each video. These multiple-choice answers serve as categorical ordinal feature vectors for a previously validated logistic regression classifier, trained on clinician-filled electronic health records, that predicts the probability that a child has autism.

For a gold standard, we use a previously published and validated [49-54] logistic regression classifier (Fig. 2), trained on electronic health record databases of autism diagnostic scoresheets filled out by expert clinicians, which emits a probability score of autism using the crowd workers’ multiple-choice responses as categorical ordinal feature vectors. Because logistic regression classifiers produce a probability, we treat the probability as a confidence score of the crowdsourced workers’ responses. We analyze the probability of the correct class (referred to as PCC), which is *p* when the true class is autism and *1-p* when the true class is neurotypical. When assessing classifier predictions, we use a threshold of 0.5. We use a worker’s average PCC for videos the worker has rated as a metric of the worker’s video tagging capability, with a higher mean PCC corresponding to greater mean performance by the worker.

### 2.4. Metrics evaluated

We strive to develop metrics which only take input parameters that do not depend on *a priori* knowledge about the correct classification score of the videos. We test the following metrics for correlation with the PCC, where *N* is the number of videos rated by a worker, *M* is the number of questions per video rating task (inputs to the diagnostic classifier), and *A_i,j,k_* is the answer for video *i* and question *j* for the *k*^*th*^ time.

#### Mean same-child L1 distance (MSCL_1_):

We asked crowd workers to rate the same child at least one month apart. Workers did not have access to their originally recorded answers and were unaware that they would be asked to rate the same video a second time when providing the first set of ratings. We observe the mean deviation for all videos between a worker’s original ratings for the video and their subsequent ratings one month later. We call this metric the *mean same-child L1 distance (MSCL_1_),* which we consider as a metric of the worker’s *test-retest reliability.* Higher values for the MSCL_1_ correspond to greater variation in worker responses when re-rating the same video one month apart. Formally, MSCL_1_ is calculated as:

$$MSCL_{1}=\frac{\sum_{i=1}^{N}\sum_{j=1}^{M}|A_{i,j,2}-A_{i,j,1}|}{N}$$

#### Mean pairwise internal L1 distance (MPIL_1_):

To analyze the reliability of the worker’s answers across videos, we look at the mean L1 distance between a worker’s answer to each video and all other videos they rated. We call this metric the *mean pairwise internal L1 distance (MPIL_1_)*. MPIL_1_ is high when workers provide a wide variety of answer patterns across videos. If the worker answers all questions the same way per video, the MPIL_1_ will be 0. Formally, MPIL_1_ is calculated as:

$$MPIL_{1}=\frac{\sum_{i1=1}^{N}\sum_{i2=1}^{N}\sum_{j=1}^{M}|A_{i2,j}-A_{i1,j}|}{0.5\;N\;(N-1)},i1<i2$$

#### Penalized time (PT):

We aimed to build a metric that prioritizes rewarding workers who spent sufficient time rating the first time while rewarding, to a lesser extent, workers who spend sufficient time rating after receiving a warning. We also aimed to penalize workers who either do not spend more time rating after receiving a warning or who do not sufficiently update their answers. We create a metric of worker *trustworthiness* taking both of these factors into account which we call the *penalized time (PT)*. If workers spend longer than a time threshold *T* rating, then they are not asked to revise their answers and receive a baseline score *M*. If they do not spend a sufficient time (*T*) rating, then they are asked to spend more time and to revise their answers. In this case, the metric consists of two terms, balanced by a weighting constant *c*. The first term is the “revision” mean same-child L1 distance (*RMSCL*_1_) between initial and revised answers only for videos that the worker was explicitly asked to revise. The second term is the mean of the total time spent rating, which is the time spent initially (*t_1_*) and the time spent revising the answers (*t_2_*). Formally, PT is calculated as:

PT={M,t1≥Tt1+t2N+cRMSCL1,t1<T}

#### Time spent:

Finally, we record the mean amount of time spent rating per video, in seconds. We hypothesized that workers who spend more time on the rating task will tend towards achieving higher performance.

We hypothesized that all four metrics are correlated with PCC. We only calculate metrics for workers who rated at least 10 videos. Because 13 questions were asked, an MSCL_1_ or MPIL_1_ of 13 means that, on average, the worker’s answer differed by 1 categorical ordinal answer choice per question (e.g., the difference between “*Mixed: some regular echoing of words and phrases, but also some language*” and “*Mostly echoed speech*” in Fig. 1).

### 2.5. Prediction of crowd worker performance from metrics

We train and test a linear regression model to predict the mean PCC of the workers using 5-fold cross validation. We evaluate all non-empty subsets of the correlative metrics described in section 2.4 as inputs to the model. Since not all workers reopened the task after receiving a warning and not all workers conducted the second task in the series, we evaluated our model both using all available workers with complete data for all metrics as well as using the subset of 55 workers with data for all metrics.

## 3. Results

### 3.1. Correlation between metrics and probability of the correct class

**Fig. 4.**
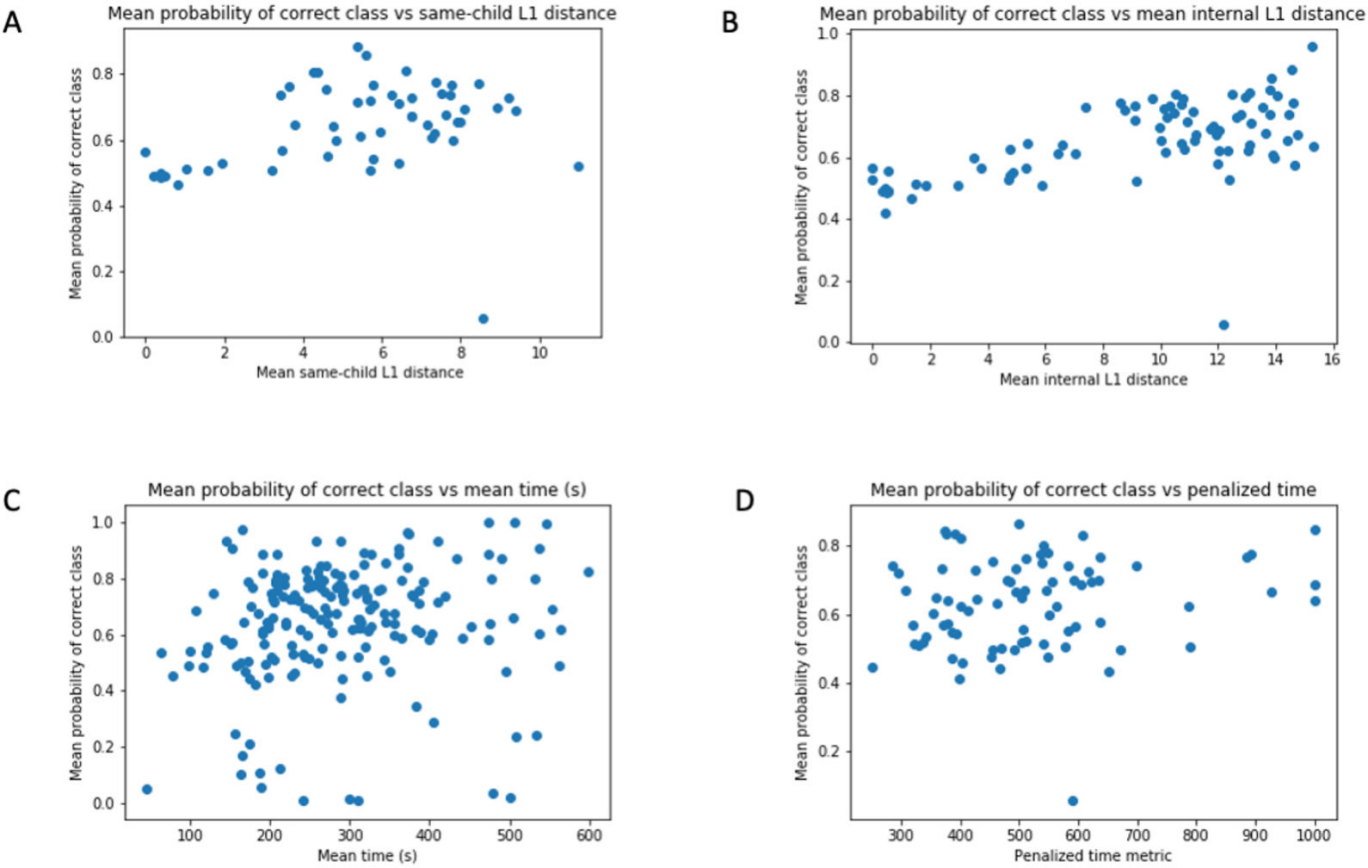
Correlations between metrics and probability of the correct class (PCC). (A) Correlation between mean same-child L1 distance and PCC. (B) Correlation between mean pairwise internal L1 distance and PCC. (C) Correlation between time spent (s) and PCC. (D) Lack of correlation between penalized time and PCC.

Correlations of each of the worker metrics with their mean PCC are displayed in Fig. 4. Mean values per worker are only plotted and analyzed if at least 5 data points are available for the worker. MSCL_1_, MPIL_1_, and mean time spent were all significantly correlated with PCC (r=0.31, p=0.0212 for MSCL_1_; r=0.57, p<0.0001 for MPIL_1_; r=0.16, p=0.0284 for time), supporting the predictive power of these metrics. Intuitively, this means that higher variability in worker answers for the same video and across videos correlates with increased worker performance. We note that only MPIL_1_ passes Bonferroni correction. Penalized time was not significantly correlated with PCC (r=0.17, r=0.1413 for penalized time).

Interestingly, Fig. 4 reveals that the presence of enough data to calculate certain metrics is in itself predictive of worker performance. Fig. 4C shows that there are several workers who had a mean PCC below 50%. However, none of these workers appear in the plot for MSCL_1_ (Fig. 4A), MPIL_1_ (Fig. 4B), or penalized time (Fig. 4D), indicating that workers with low average performance did not rate videos again after one month and did not revise their answers when prompted.

**Fig. 5.**
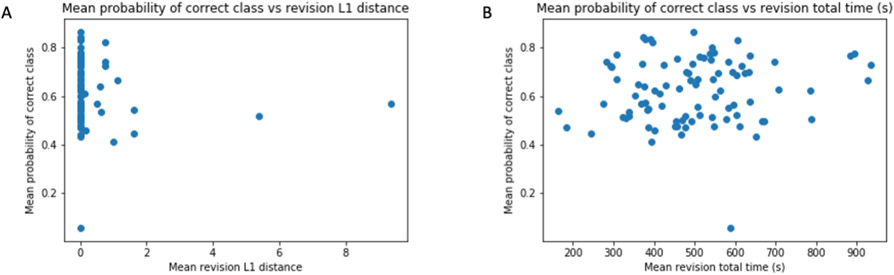
Lack of correlation between PCC and (A) the total time spent rating post-warning and (B) the L1 distance between the answer before and after the warning.

We evaluate all values of the weighting constant *c* for the penalized time metric in the interval [0.05, 10.0] using a step size of 0.05. No value resulted in a metric that positively correlates with PCC. To investigate, we review the correlation between both terms of penalized time: (1) the mean total time spent rating post-warning and (2) the mean L1 distance between the answer vector before and after the warning (Fig. 5). Neither of these metrics are correlated with PCC (r=−0.10, p=0.3414 for revision L1 distance; r=0.11, p=0.2908 for total time), explaining the inability of the penalized time metric to predict PCC regardless of the parameters chosen.

### 3.2. Regression prediction of the mean probability of the correct class

Table 1 contains the mean average error (MAE) of a linear regression model predicting the probability of the correct class for each worker using metrics on the same set of videos. There were 55 workers with data for all 3 metrics used in the regression model. For these workers, all metrics predicted the PCC with less than 10% MAE.

The MAE when using all 3 features performs nearly identically, to two decimal places, compared to using only MSCL_1_ and MPIL_1_. Mean time does not contribute much predictive power given the other metrics. Interestingly, the most predictive input configuration when using the same 55 workers is MPIL_1_ together with mean time (6.97% MAE), followed by MPIL_1_ alone as a close second (6.98% MAE). This is a testament to the success of the MPIL_1_ metric.

Table 2 contains the mean average error of a linear regression model predicting the probability of the correct class for each worker using metrics from one set of children and mean probability of the correct class calculations for a distinct set of children. The most predictive input feature configuration (MSCL_1_ and MPIL_1_) results in a MAE of 10.41%, only 3.44% higher than the best MAE when training and testing on the same set of videos and workers using cross-validation (Table 1). MPIL_1_ is involved in all of the top-4 input metric configurations resulting in the lowest MAE, again verifying the success of the MPIL_1_ metric.

## 4. Discussion and Future Work

We identify three metrics which are individually highly correlated with the mean probability of the worker’s categorical behavioral feature tags predicting the correct class. In particular, one of our two reliability metrics - the mean pairwise internal L1 distance, which is the mean L1 distance between a worker’s answer to each video and all other videos they rated - stood out as the most predictive metric. Mean pairwise internal L1 distance alone can predict a worker’s PCC within 7% MAE when trained on the same set of workers as in the test set but with different videos, and it can predict PCC within 11% MAE when trained on one group of workers and tested on an entirely district set of workers and videos. This metric alone therefore provides a powerful behavioral predictor of worker performance and is therefore likely to be useful for rapidly filtering workers. The positive correlation shown in Fig. 4B suggests that unreliable workers will provide the same or similar patterns of answer sequences for each task. We see that an increasing diversity of answers between tasks results in a higher PCC for the entire spectrum of possible L1 distances. Intuitively, this may be a result of the diverse set of features exhibited by the heterogeneous behavioral characteristics of the children in our dataset.

Interestingly, the raw time metric is not particularly correlative with PCC, indicating that analyzing the answer domain is more informative than the time domain. For workers who received a warning for low time spent, neither the time spent revising post-warning nor the L1 distance between the original and revised set of answers was predictive of the workers’ final performance. It is possible that once workers are aware that their time is tracked, they idly keep the rating interface open, accumulating time without accumulating thoughtful work. This hypothesis is speculative, and more fine-grained timing information must be recorded to evaluate such hypotheses.

Future work should evaluate workers on a larger scale, which will validate the preliminary findings of the present study. It is possible that predictive time-based trustworthiness metrics exist. Evaluation on a larger scale in conjunction with more fine-tuned worker metrics will lead to more precise predictions.

## 5. Conclusion

We demonstrate that behavioral metrics about crowd workers can predict, with a high degree of accuracy, the performance of crowd workers on behavioral feature extraction tasks for the binary diagnosis of autism. Metrics like these can be used for quickly and efficiently identifying crowd workers who are trustworthy and reliable enough for exposure to highly sensitive PHI based on a quantification of their reliability.

## Figures and Tables

**Fig. 3.**
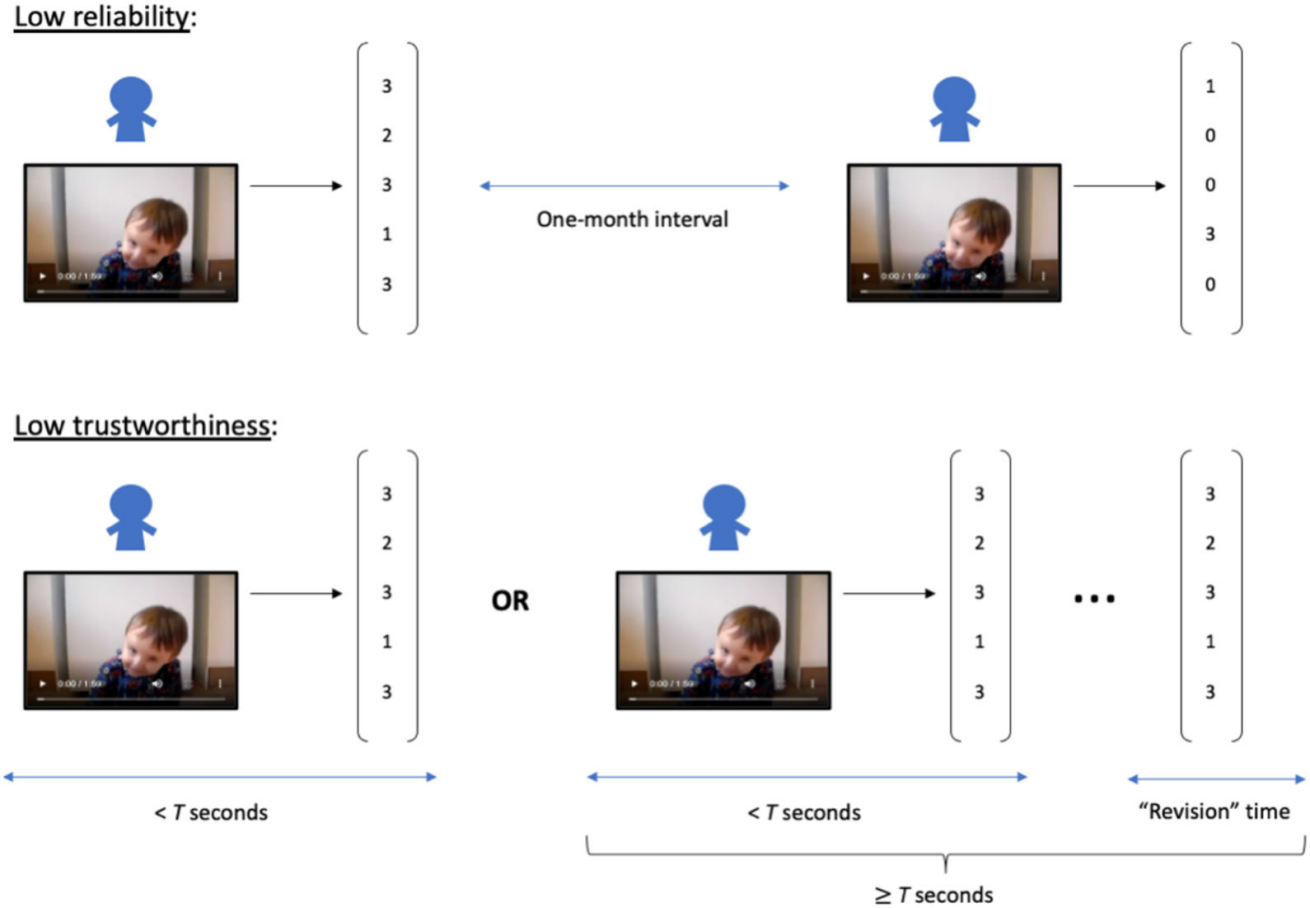
Process for calculating trust and reliability metrics for crowd workers. The reliability of workers is determined by how different their answers are when rating the same video one month apart. The trustworthiness of workers is determined by whether they spend the minimal amount of time needed to properly answer the questions, whether they spend sufficient time when receiving a warning, and whether their original answers change after receiving the warning.

**Table 1.** 5-fold cross validated mean average error (MAE) of a linear regression model predicting the probability of the correct class for each worker using metrics on the same set of videos.

Input Features	5-fold MAE (%) All data points	5-fold MAE (%) 55 workers with all metric data	N
MSCL_1_, MPIL_1_, mean time	7.51	7.51	55
MSCL_1_, mean time	8.89	8.89	55
MPIL_1_, mean time	7.43	6.97	81
MSCL_1_, MPIL_1_	7.51	7.51	55
MSCL_1_	9.24	9.24	55
MPIL_1_	7.39	6.98	81
Mean time	15.56	9.83	193

**Table 2.** Mean average error (MAE) of the linear regression model predicting the probability of the correct class for each worker using the same metric data and resulting classifier weights *for the workers and videos used in* Table 1 and mean probability of the correct class calculations for a *distinct set of videos* for a *distinct set of workers*.

Input Features	MAE (%) All data points
MSCL_1_, MPIL_1_, mean time	10.93
MSCL_1_, mean time	13.03
MPIL_1_, mean time	11.50
MSCL_1_, MPIL_1_	10.41
MSCL_1_	11.87
MPIL_1_	10.91
Mean time*	12.10

* Mean time as the only feature is the only configuration of input features that requires a different set of data points: N=102 instead of a subset of size N=62 for all other configurations.
